# Intracardiac Echocardiography and Coronary Angiography During Ventricular Ablation From the Aortic Cusps and Commissures: Marked Inter‐Center Variability Without an Observed Coronary Injury Signal

**DOI:** 10.1111/jce.70383

**Published:** 2026-05-26

**Authors:** Dakota De Cecco, Jeanne du Fay de Lavallaz, Henry D. Huang, Boldizsar Kovacs, Jennifer Beney, Tobias Reichlin, Patrick Badertscher, Fabian Jordan, Christian Sticherling, Jackson J. Liang

**Affiliations:** ^1^ Electrophysiology Section, Division of Cardiology University of Michigan Ann Arbor Michigan USA; ^2^ Electrophysiology Section, Division of Cardiology Rush University Medical Center Chicago Illinois USA; ^3^ Electrophysiology Section, Division of Cardiology University of Chicago Chicago Illinois USA; ^4^ Electrophysiology Section, Division of Cardiology Inselspital, University Hospital Bern Bern Switzerland; ^5^ Electrophysiology Section, Division of Cardiology University Hospital Basel Basel Switzerland

**Keywords:** aortic cusps, catheter ablation, coronary angiography, intracardiac echocardiography, outflow tract arrhythmias, premature ventricular complexes, ventricular tachycardia

## Abstract

**Introduction:**

Catheter ablation of premature ventricular complexes (PVCs) and ventricular tachycardia (VT) arising from the aortic cusps and commissures is effective but performed near the coronary ostia, raising concern for coronary injury. Adjunct imaging with intracardiac echocardiography (ICE) and coronary angiography is variably used, and its incremental benefit remains uncertain.

**Methods:**

We analyzed 311 ablation procedures performed in 290 patients across four high‐volume centers in the United States and Switzerland. Imaging strategies were categorized as no adjunct imaging, ICE only, angiography only, or combined ICE plus angiography.

**Results:**

Marked inter‐center variability in imaging use was observed. Procedure duration and fluoroscopy exposure differed significantly by imaging strategy and were greatest when both ICE and angiography were used (*p* < 0.001). Acute elimination of targeted PVCs occurred in 87% of procedures. No coronary artery injury was observed.

**Conclusion:**

Adjunct imaging strategies for aortic cusp and commissure PVC/VT ablation vary widely across centers and substantially influence procedural efficiency. Despite frequent ablation near the coronary ostia, no coronary injury events were observed.

## 1 Introduction

Catheter ablation of premature ventricular complexes (PVCs) and ventricular tachycardia (VT) arising from the aortic cusps and cusp commissures is an effective treatment strategy for symptomatic outflow tract arrhythmias [1]. These procedures require radiofrequency energy delivery in close anatomic proximity to the coronary ostia and the aortic valve apparatus, raising concern for potential coronary injury or valvular compromise [2, 3]. To mitigate the perceived risk of coronary injury, operators frequently employ adjunctive imaging strategies—most commonly intracardiac echocardiography (ICE), coronary angiography, or both. However, the extent to which these imaging approaches are used in routine practice varies widely, and their incremental safety benefit remains incompletely defined.

We evaluated center‐level variability in imaging strategy and associated procedural and safety outcomes in a multicenter cohort of patients undergoing aortic cusp or commissure PVC/VT ablation.

## 2 Patient Characteristics

The cohort comprised 290 patients undergoing 311 ablation procedures at four high‐volume centers (two in the United States and two in Switzerland; University Hospital of Michigan, Ann Arbor, USA; Rush University Medical Center, Chicago, USA; and Inselspital, University Hospital Bern, University Hospital, Basel, Switzerland) between 2015 and 2024. To avoid direct comparison of institutional practices, centers are presented in de‐identified fashion (Center 1–4) in figures and results. Median age was 65 years, and 26% were women. Structural heart disease was common (62% cardiomyopathy; 35% history of heart failure), with coronary artery disease present in 24%. The primary indication was symptomatic PVCs (92%), and 22% had sustained VT.

Across the 311 procedures, 88% were PVC ablations, and 21% involved VT, with 72% performed as first‐time procedures. Ablation was performed in the aortic cusps in 76% of procedures and in the cusp commissures in 42%, most commonly at the right–left commissure. Imaging strategy was categorized as follows: (1) neither ICE nor coronary angiography, (2) ICE only, (3) angiography only, or (4) ICE plus angiography.

## 3 Marked Inter‐Center Variability in Imaging Strategy

Imaging practice differed substantially across centers. Centers 1 and 2 more frequently performed cusp and commissure ablation without routine adjunct imaging, relying on fluoroscopic landmarks and electroanatomic mapping. In contrast, Center 3 predominantly employed ICE‐driven workflows, including both ICE only and combined ICE plus angiography approaches. Center 4 demonstrated a mixed strategy, employing angiography selectively. This striking heterogeneity highlights the absence of a convergent standard of care and suggests that imaging strategy is primarily influenced by institutional culture, laboratory infrastructure, and operator preference. Notably, even current expert consensus guidance stops short of mandating routine coronary angiography for all cusp and commissure ablations, instead recommending its consideration based on anatomic proximity, a recommendation that inherently permits the wide practice variation observed in our cohort [4].

## 4 Procedural Efficiency Trade‐Offs

Procedure duration varied significantly by imaging strategy (Table 1). Median procedure time was shortest when neither ICE nor angiography was used (~110 min) and longest when both ICE and angiography were employed (~269 min), with intermediate durations for ICE only (~208 min) and angiography only (~122 min) approaches (*p* < 0.001).

**Table 1:** Procedural characteristics and outcomes according to adjunct imaging strategy during aortic cusp and commissure PVC/VT ablation.

Variable	Neither (*n* = 67)	ICE only (*n* = 123)	Angiography only (*n* = 20)	ICE + angiography (*n* = 92)	*p* value
*Procedural efficiency*					
Procedure time, min	110 (94–159)	208 (154–297)	122 (99–150)	269 (200–358)	< 0.001
Fluoroscopy time, min	4 (1–6)	12 (6–20)	4 (2–9)	26 (16–38)	< 0.001
PVC morphologies targeted	1 (1–2)	2 (2–4)	2 (1–2)	2 (2–5)	< 0.001
*Acute procedural success*					
Complete PVC elimination	49 (80%)	86 (95%)	17 (89%)	60 (82%)	0.020
Partial success	9 (15%)	4 (4%)	2 (11%)	13 (18%)	
Failure	3 (5%)	1 (1%)	0	0	
*Coronary angiography findings*					
Pre‐ablation abnormalities	—	—	7 (47%)	1 (13%)	0.20
Post‐ablation abnormalities	—	—	0	8 (9%)	> 0.9
*Clinical outcomes*					
Coronary injury	0	0	0	0	—

*Note:* Procedural duration and fluoroscopy exposure increased substantially with more intensive imaging strategies, particularly when both ICE and coronary angiography were used. Acute procedural success was high across all strategies, and no coronary artery injury was observed regardless of imaging approach. Values are median (interquartile range) or *n* (%). *p*‐Values represent comparisons across the four groups using chi‐square tests for categorical variables and Kruskal–Wallis tests for continuous variables.

Notably, the use of ICE did not translate into reduced fluoroscopy exposure. Fluoroscopy time was lowest in procedures without adjunct imaging (~4 min) and progressively higher with ICE only (~12 min) and ICE plus angiography (~26 min) strategies (*p* < 0.001). This likely reflects, at least in part, increased procedural complexity and longer case duration in patients selected for more intensive imaging rather than a direct causal effect of imaging modality itself.

## 5 Anatomic Targets and Imaging Utilization

Imaging uses closely tracked anatomic complexity. ICE and angiography were more frequently employed in procedures involving cusp commissures, particularly the right–left commissure, where the proximity of the left main coronary artery ostium to the ablation target heightens the perceived risk of coronary injury [4, 5]. In contrast, ablations limited to a single cusp—especially outside the commissural regions—were more often performed without adjunct imaging. Prior single‐center experience has suggested that routine coronary angiography may be unnecessary for many aortic cusp ablations, although these studies included limited commissural targets [6, 7]. Our multicenter data extend these findings by demonstrating a similarly favorable safety profile even in the substantial proportion of procedures targeting the cusp commissures.

## 6 Safety Outcomes: No Observed Coronary Injury Signal

Across all 311 ablations, no coronary artery injury was observed, irrespective of imaging strategy. This finding is consistent with the observation from experienced operators that coronary injury is uncommon during cusp ablation, particularly when energy is delivered at the base of the cusps [5]. This safety signal held even in a substantial proportion of procedures involving commissural targets, where the anatomic proximity to the coronary ostia is closest. In the subset of procedures where coronary angiography was performed, abnormalities were reported in 38% of pre‐ablation angiograms (8 of 16) and 10% of post‐ablation angiograms (8 of 78). These findings likely reflect a heterogeneous mixture of pre‐existing coronary disease, transient catheter‐related ostial changes, or angiographic variants rather than clinically meaningful ablation‐related injury. Importantly, none of these angiographic findings were associated with documented coronary complications or adverse clinical sequelae.

Acute ablation efficacy was high, with complete elimination of targeted PVCs achieved in 80%, 95%, 89%, and 82% of cases across the four groups, respectively (*p* = 0.020). Notably, procedures performed with ICE alone were associated with the highest rate of PVC elimination. During a median follow‐up of approximately 3.5 years, recurrence occurred in 21%, most commonly due to PVCs or VT with morphology similar to the index arrhythmia.

## 7 Conclusions

**Figure 1:**
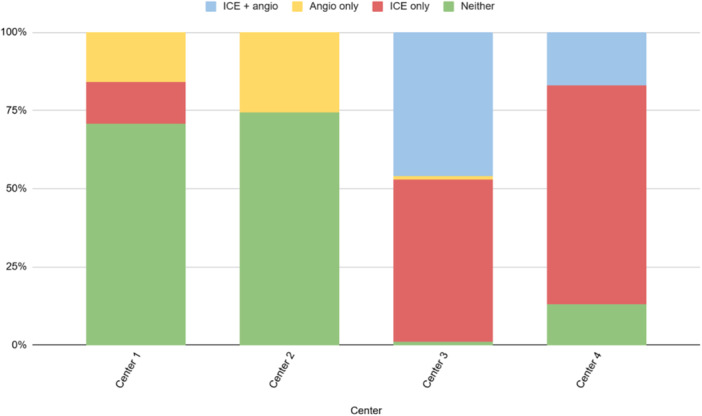
Inter‐center variation in adjunct imaging use during aortic cusp and commissure ablation. Stacked bars display the proportion of procedures at each participating center performed with the following imaging strategies: combined intracardiac echocardiography (ICE) and coronary angiography, angiography alone, ICE alone, or neither modality. Centers are shown in de‐identified fashion (Centers 1–4) to emphasize variability in imaging strategies rather than direct institutional comparison. These data illustrate substantial variability in imaging approach across centers.

In summary, our multicenter analysis of aortic cusp and commissure PVC/VT ablation highlights pronounced variability in adjunct imaging practice for aortic cusp and commissure PVC/VT ablation, which is shaped primarily by institutional tradition and operator preference. This heterogeneity was accompanied by substantial differences in procedural duration and fluoroscopy exposure, with more intensive imaging strategies markedly increasing both. Acute procedural success was high across all imaging approaches, with the highest rates of PVC elimination observed in procedures utilizing ICE alone. Importantly, despite these varied workflows, and frequent ablation near the coronary ostia, no coronary injury events occurred in over 300 ablations. These findings support the overall feasibility and safety of cusp and commissure ablation across diverse real‐world workflows, and challenge the assumption that routine use of coronary angiography is necessary for safety. Future studies should focus on identifying patient, anatomic, and lesion‐specific features that may derive the greatest benefit from routine ICE, coronary angiography, or standardized hybrid imaging approaches (Figure 1).

## Funding

The authors have nothing to report.

## Conflicts of Interest

The authors declare no conflicts of interest.

## Data Availability

The data that support the findings of this study are available from the corresponding author upon reasonable request.
